# Heterostructure ReS_2_/GaAs Saturable Absorber Passively Q-Switched Nd:YVO_4_ Laser

**DOI:** 10.1186/s11671-019-2953-7

**Published:** 2019-03-29

**Authors:** Lijie Liu, Hongwei Chu, Xiaodong Zhang, Han Pan, Shengzhi Zhao, Dechun Li

**Affiliations:** School of Information Science and Engineering, Shandong University, Qingdao, 266000 China

**Keywords:** Q-switching lasers, Two-dimensional nanomaterials, Saturable absorbers

## Abstract

Heterostructure ReS_2_/GaAs was fabricated on a 110-μm (111) GaAs wafer by chemical vapor deposition method. Passively Q-switched Nd:YVO_4_ laser was demonstrated by employing heterostructure ReS_2_/GaAs as a saturable absorber (SA). The shortest pulse width of 51.3 ns with a repetition rate of 452 kHz was obtained, corresponding to the pulse energy of 465 nJ and the peak power of 9.1 W. In comparison with the ReS_2_ Q-switched laser and the GaAs Q-switched laser, the heterostructer ReS_2_/GaAs Q-switched laser can generate shorter pulse duration and higher pulse energy.

## Introduction

Passive Q-switching technologies have been extensively applied in industry, medical science, and scientific research because of its noticeable advantages with respect to simple structure and considerable efficiency [1–4]. Various materials have been used as saturable absorbers, in which the most common one is the semiconductor saturable absorber [5–7]. Compared with SESAM, two-dimensional (2D) materials show great potential owing to the broad bandwidth, low cost, and easy fabrication. In recent years, 2D materials like black phosphorus, graphene, and transition mental dichalcogenides (TMDs), have been widely adopted as SAs in the passive Q-switching lasers [8–12]. Among these reported TMDs, such as MoS_2_, MoSe_2_, and WS_2_, one characteristic is its indirect-to-direct bandgap change occurs when going from bulk to monolayer [13, 14].

Unlike those abovementioned TMDs, ReS_2_ has a direct bandgap, whose value remains ~ 1.5 eV in both bulk and monolayer forms [15]. Furthermore, the photoelectric properties of ReS_2_ are similar from bulk to monolayer [16]. As a semiconductor, ReS_2_ exhibits strong nonlinear absorption, so that ReS_2_ as SA has been experimentally used in solid lasers in 1.5-μm, 2.8-μm, and 3-μm wavelength [17–19]. Recently, ReS_2_ based on sapphire substrate has been reported as a saturable absorber in 1-μm laser [20]. However, the ReS_2_ saturable absorber was adhered to the sapphire substrate with the weak van der Waals forces, which is easily cleaved from the substrate [20]. Up to date, GaAs has been generally applied in Nd-doped solid-state lasers for Q-switching at 1 μm [21]. However, GaAs can also be combined with other semiconductors into heterostructures, such as MoS_2_/GaAs, MoSe_2_/GaAs, and PtSe_2_/GaAs [22]. So far, the heterostructure semiconductor MoS_2_/GaAs SA has been used to get shorter pulses [23], convincing us that the similar heterostructure could be attractive for the pulsed operation. The chemical vapor deposition (CVD) technology can precisely control the deposition thickness and generate cleanly lattice-matched surface. In comparison with the ReS_2_ on sapphire substrate, semiconductor ReS_2_/GaAs heterostructures as quantum well can confine the carrier and greatly improve the population inversion. The performance of the heterostructure ReS_2_/GaAs saturable absorber could be expected.

In this paper, the heterostucture semiconductor ReS_2_/GaAs is firstly fabricated. As saturable absorber, a passively Q-switched Nd:YVO_4_ solid-state laser was demonstrated with heterostucture ReS_2_/GaAs. In comparison with the ReS_2_ saturable absorber or GaAs semiconductor saturable absorber, the laser performance was greatly enhanced with the heterostucture ReS_2_/GaAs saturable absorber. The experimental results reveal that the ReS_2_/GaAs saturable absorber could be of great interest for passive Q-switching operation.

## Methods/Experimental

**Fig. 1:**
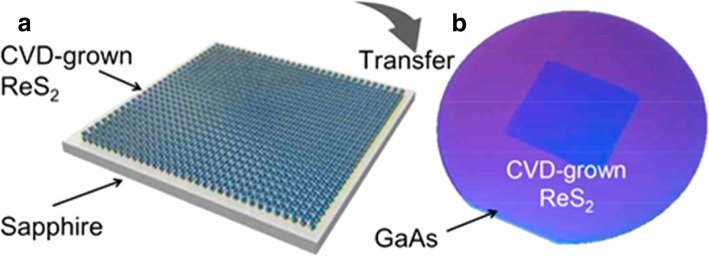
**a**, **b** The fabrication procedure of the ReS_2_/GaAs heterostructure

Recently, the ReS_2_ saturable absorber is prepared by liquid phase exfoliation (LPE) owing to the low cost. However, ReS_2_ monolayer in our experiment was synthesized by CVD because we can precisely control the thickness of ReS_2_. Here, sulfur powder and ammonium perrhenate (NH_4_ReO_4_) were used as the precursors for growth. The ReS_2_ monolayer was grown on a clean sapphire wafer. During the deposition process, argon was employed as the carrier gas for sulfur. Then, we transferred the CVD grown ReS_2_ monolayer to a 110-μm-depth GaAs wafer with a dimension of 10 × 10 mm^2^ to make up the heterostructure. The total procedure was shown in Fig. 1.

**Fig. 2:**
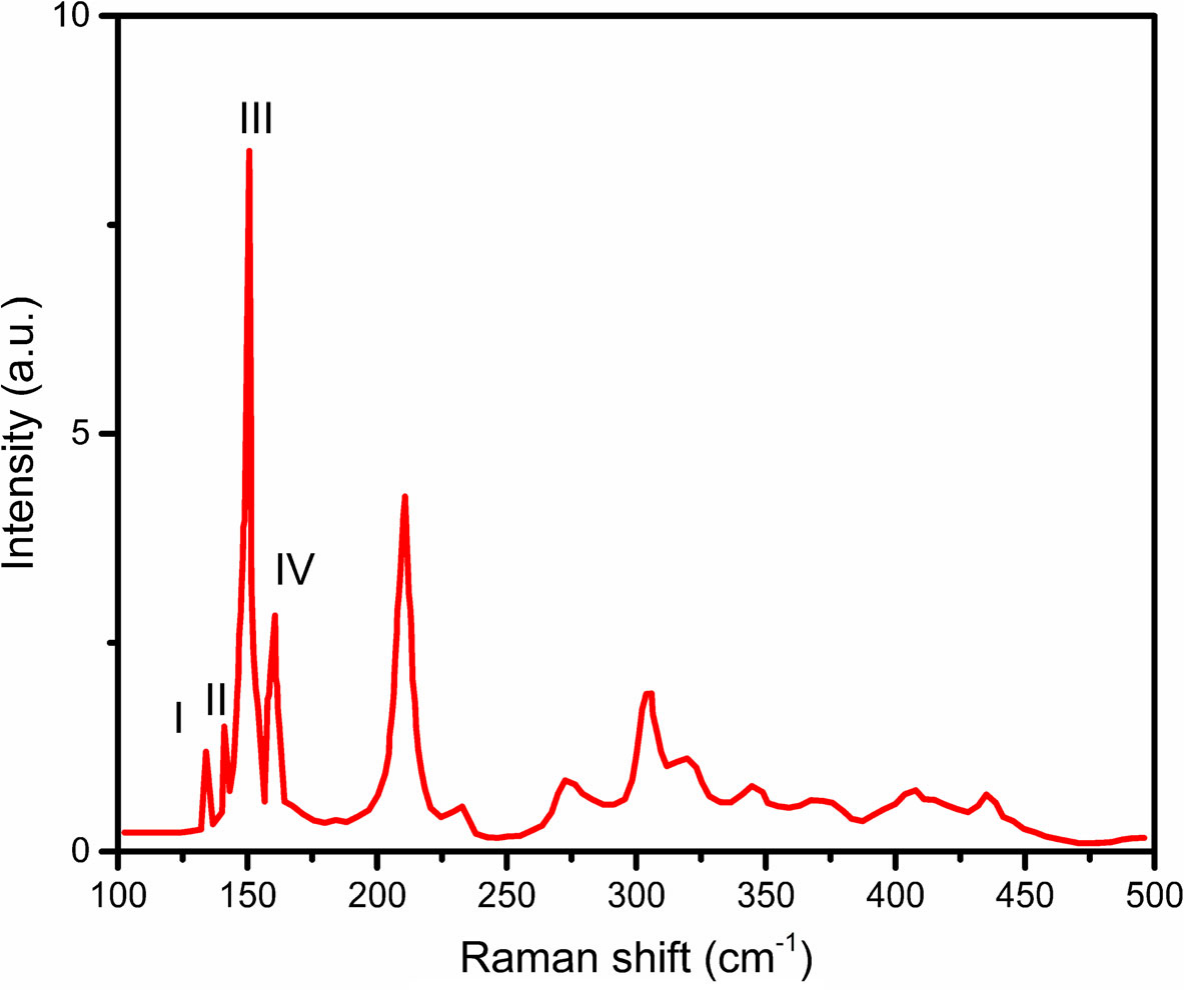
Raman spectroscopy of the heterostructure ReS_2_/GaAs

To make sure the layer number of the prepared ReS_2_/GaAs heterostructure, we investigated the Raman shift of the prepared sample (Fig. 2). The *A*_*g*_ modes located at 134 and 141 cm^−1^, while the *E*_*g*_ modes located at 150.7, 160.6, 210.7, and 233 cm^−1^. The difference of III-I peaks was 16.7 cm^−1^, which was considered as monolayer [24].

**Fig. 3:**
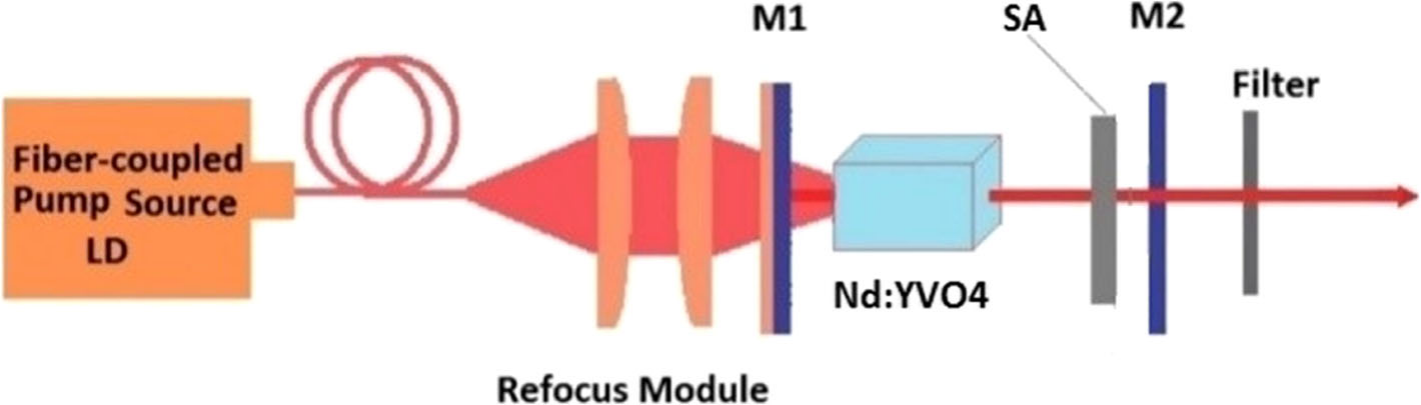
Schematic of the Q-switching laser cavity

Figure 3 shows the schematic of the passively Q-switched laser with the ReS_2_/GaAs heterostructure saturable absorber. A 0.1%-Nd-doped c-cut Nd:YVO_4_ was employed as the laser crystal, whose dimensions were 3 × 3 × 10 mm^3^. The passively Q-switched laser was end-pumped by a fiber-coupled diode laser at 808 nm. The pump beam was then focused into the crystal with a refocus module with a spot on the gain medium with 400-μm in diameter. A concave mirror M1 was used as the input mirror, which had antireflection (AR) coating at 808 nm on two sides and high-reflection (HR) coating at 1064 nm inside the resonator. The curvature radius of M1 was 200 mm. A flat mirror M2 worked as output coupler (OC) with the transmission at 1064 nm of 10%. A short and linear cavity with a length of about 30 mm was formed. The ReS_2_/GaAs (or GaAs) was then inserted into the cavity working as saturable absorber and put near the output coupler.

## Results and Discussion

**Fig. 4:**
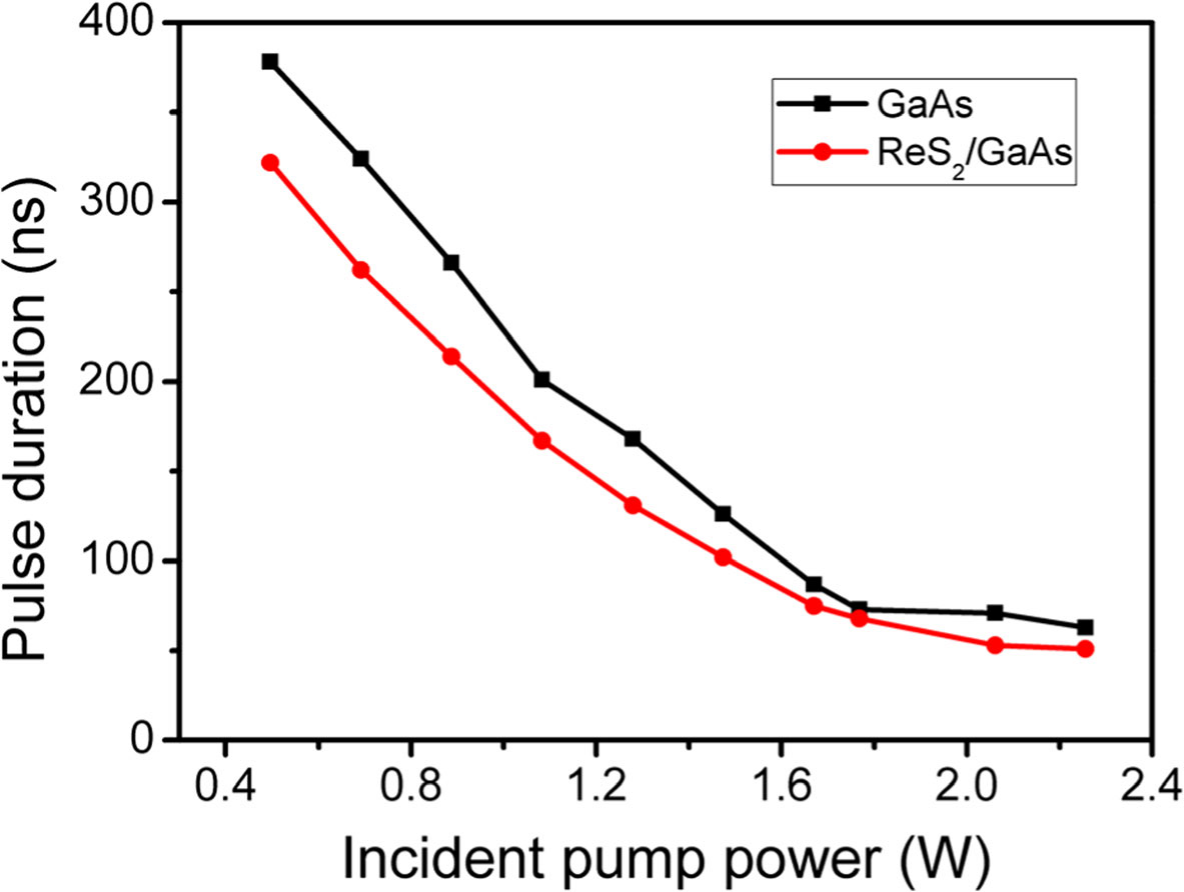
Pulse duration of the Q-switched laser versus the incident pump power

**Fig. 5:**
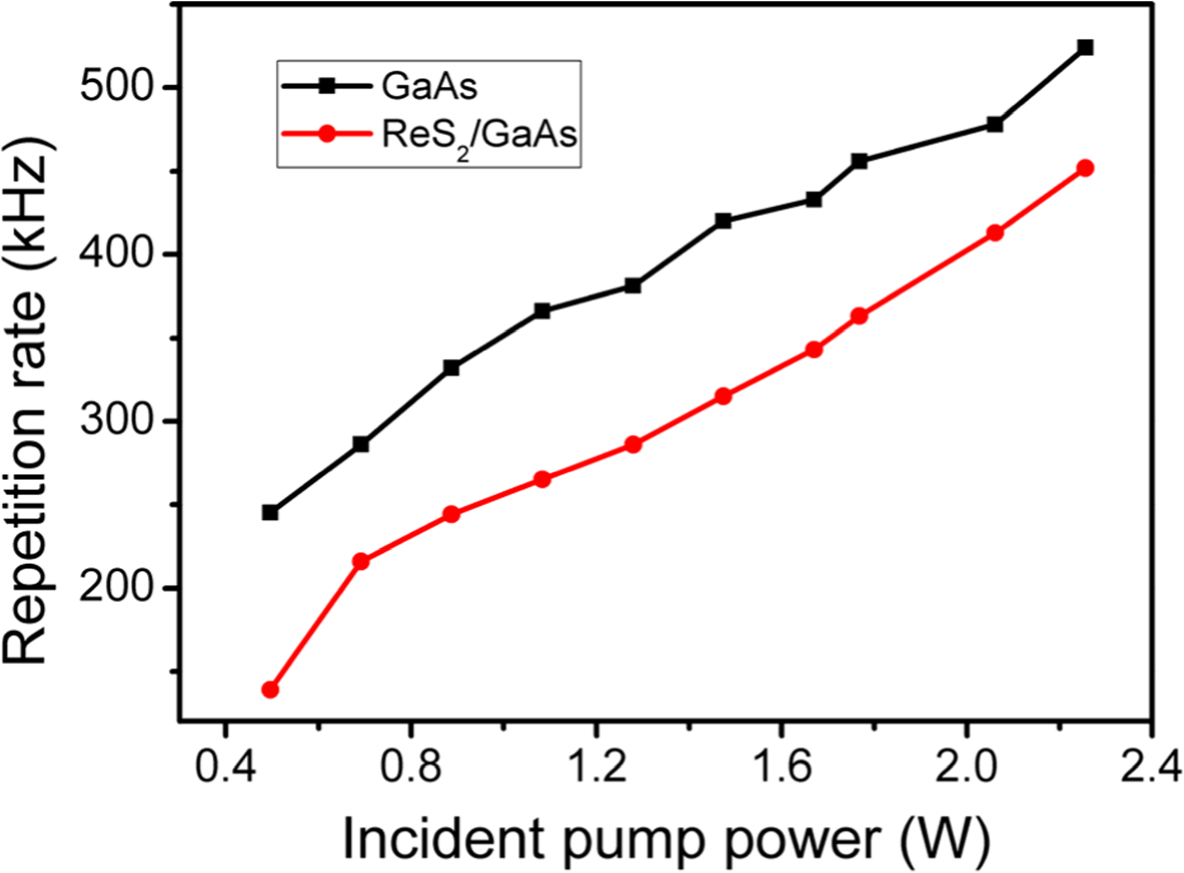
Repetition rate of the passively Q-switched laser versus the incident pump power

The pulse duration and repetition rate were recorded with a digital phosphor oscilloscope (DPO 7104C) via a fast InGaAs photodiode. As shown in Fig. 4 and Fig. 5, with increasing the input power from 0.5 to 2.26 W, the pulse duration from the ReS_2_/GaAs passively Q-switched laser decreased from 322 to 51.3 ns, while the repetition rate increased from 139 to 452 kHz. In comparison, we also set up the GaAs Q-switched laser. We can see from Figs. 4 and 5 that the ReS_2_/GaAs heterostructure is contributed to shortening the pulse width and lower the pulse repetition rate.

**Fig. 6:**
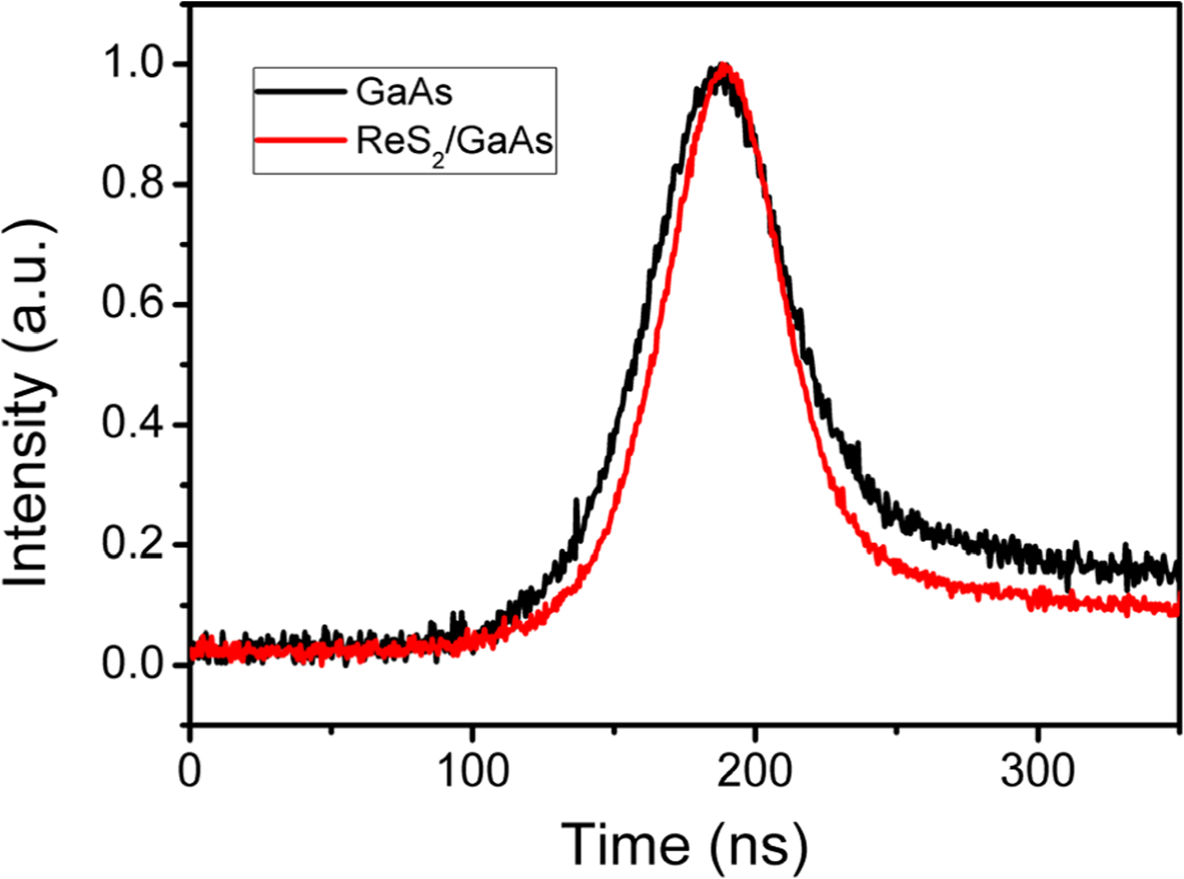
Profile of the Q-switching laser based on ReS_2_/GaAs or GaAs at the incident pump power of 2.26 W

Figure 6 shows the profiles of Q-switching pulses at the pump power of 2.26 W with different semiconductor saturable absorbers. The output pulses with the pulse width of 51.3 ns and the pulse energy of 465 nJ can be achieved with the ReS_2_/GaAs heterostructure saturable absorber. In contrast, the output pulse duration from the GaAs Q-switched laser was 63.2 ns with the pulse energy of 435 nJ, which was shown in the inset picture. Figure 6 also implies that the symmetry of the ReS_2_/GaAs Q-switched pulse is comparatively much better.

**Fig. 7:**
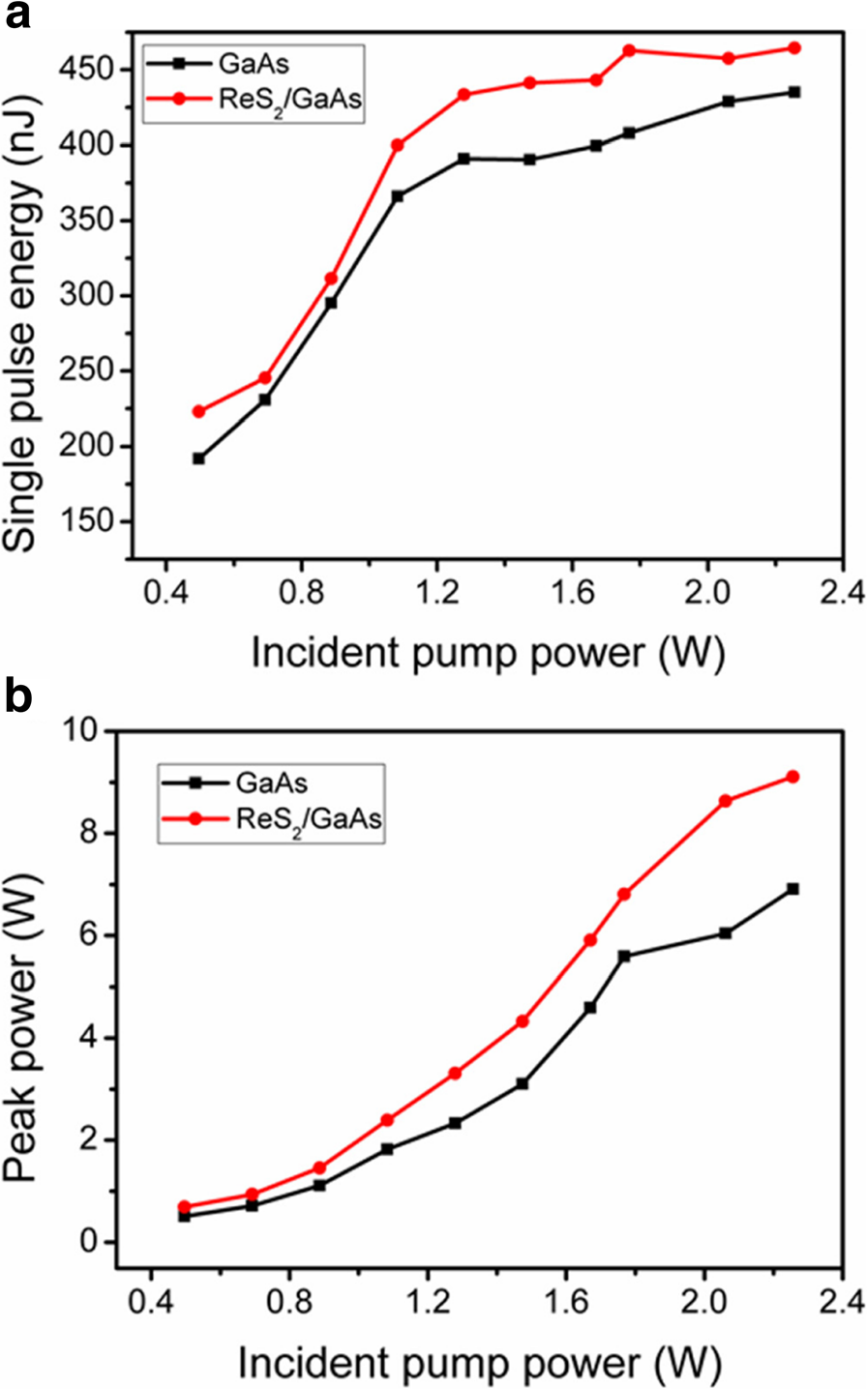
Pulse energy (**a**) and peak power (**b**) of the Q-switching laser

The pulse energy and peak power versus the incident pump power are demonstrated in Fig. 7. With increasing pump power, there was a rapid increase in peak power. In addition, the peak power and pulse energy of the ReS_2_/GaAs Q-switched laser are higher than those of GaAs-based Q-switched laser at the same conditions. And for ReS_2_/GaAs Q-switched laser, the maximum peak power of 9.1 W and the highest pulse energy of 465 nJ can be achieved at 2.26 W pump power.

We also compared our experimental results with the previous work [20] with the ReS_2_ saturable absorber on the sapphire substrate. The shortest pulse duration from the ReS_2_ Q-switched 1-μm laser was 139 ns with a repetition rate of 644 kHz, corresponding to a peak power of 1.3 W. As a consequence, the heterostucture ReS_2_/GaAs saturable absorber can obviously improve the laser performance, especially in terms of pulse duration, pulse energy, and peak power, when compared with the ReS_2_ Q-switched lasers or GaAs Q-switched lasers.

## Conclusions

In summary, the heterostructure ReS_2_/GaAs saturable absorber was first fabricated. Based on the ReS_2_/GaAs heterostructure saturable absorber, the passively Q-switched Nd:YVO_4_ laser was demonstrated. At the pump power of 2.26 W, the minimum pulse duration of 51.3 ns with a repetition rate of 452 kHz was achieved, corresponding to the highest pulse energy of 465 nJ and the peak power of 9.1 W. Our results confirm that the heterostucture ReS_2_/GaAs is beneficial to improving the Q-switching performance in comparison with the semiconductor ReS_2_ or GaAs saturable absorbers.

## Abbreviations

2D: Two-dimensional
AR: Antireflection
CVD: Chemical vapor deposition
HR: High reflection
LPE: Liquid phase exfoliation
OC: Output coupler
SESAM: Semiconductor saturable absorber mirror
TMD: Transition mental dichalcogenide
